# Understanding school staff members’ enforcement of school tobacco policies to achieve tobacco-free school: a realist review

**DOI:** 10.1186/s13643-019-1086-5

**Published:** 2019-07-19

**Authors:** Anu Linnansaari, Michael Schreuders, Anton E. Kunst, Arja Rimpelä, Pirjo Lindfors

**Affiliations:** 1 Faculty of Social Sciences, Health Sciences, Tampere University, P.O. Box 100, 33014 Tampere, Finland; 2 Department of Public Health, Amsterdam UMC, University of Amsterdam, Amsterdam Public Health Institute, Amsterdam, The Netherlands; 3 PERLA—Tampere Centre for Childhood, Youth and Family Research, Tampere University, 33014 Tampere, Finland; 4 Department of Adolescent Psychiatry, Pitkäniemi Hospital, Tampere University Hospital, 33380 Nokia, Finland

**Keywords:** School tobacco policies, Implementation, Enforcement, School staff members, Realist review

## Abstract

**Background:**

School tobacco policies (STPs) that aim to achieve a tobacco-free environment require consistent enforcement by school staff. However, little is known about why staff choose whether or not to enforce STPs. Therefore, we investigated staff members’ responses to STPs that determine enforcement. Furthermore, we examined how these responses depend on contextual factors at the individual, interpersonal, school, implementation, and national levels.

**Methods:**

We performed a realist review (RR), which synthesizes existing primary evidence into a programme theory demonstrating key causal pathways through Context-Mechanism-Outcome configurations (CMOs). These CMOs link contextual factors to outcomes (i.e. staff enforcement) by explaining the underlying generative mechanisms (i.e. staff members’ cognitive, psychosocial, and behavioural responses). A systematic literature search for the period 2000–2016 was performed using Academic Search Premier, PsycInfo, and MEDLINE. Forty English-language articles were identified for the synthesis.

**Results:**

Our programme theory demonstrated three CMOs: when contextual factors make staff members experience STP enforcement as part of their professional role and duties, it may lead to staff members showing responsibility for STP enforcement (CMO1); when contextual factors make staff members feel their contribution is leading to positive outcomes, it may lead to staff members showing motivation to enforce STPs (CMO2), and when contextual factors make staff members feel that they are able to deal with students’ responses, it may lead to staff members showing confidence in STP enforcement (CMO3). Moreover, the programme theory provided more precise insights into what contextual factors contribute to triggering the individual mechanisms and the consequent outcomes.

**Conclusions:**

By applying a realist approach, we have been able to detect three CMOs explaining staff members’ STP enforcement. The findings provide useful insights explaining how stakeholders can support staff members’ STP enforcement and consequently improve the impact of STPs on adolescent smoking.

**Electronic supplementary material:**

The online version of this article (10.1186/s13643-019-1086-5) contains supplementary material, which is available to authorized users.

## Background

School tobacco policies (STPs) aim to decrease adolescent smoking behaviour and exposure to second-hand smoke by restricting smoking only to certain areas or banning smoking completely in the school buildings and outside premises during school hours. The rationale for STPs is based on research evidence showing that (i) the onset of smoking usually occurs in adolescence, (ii) schools have a major influence on adolescent smoking uptake, and (iii) schools are significant settings for health promotion [1]. Moreover, STPs receive wide public support [2] and are considered an essential element in the tobacco de-normalizing process with the aim of making the future smoke-free [3].

Research shows that STPs effectively decrease adolescents’ exposure to second-hand smoke [4–6], but evidence about their impact on smoking behaviour remains inconclusive [7, 8]. Reviews have explained the conflicting evidence by highlighting the differences in the implementation of STPs, something that most studies have not adequately taken into account [7, 8]. Implementation refers to the process of integrating and enforcing new practices within a setting [9]. A key element of implementation that improves the effectiveness of STPs on adolescent smoking behaviour is strict and consistent enforcement by school staff members [7, 8, 10]. According to Schreuders et al. [10], strict and consistent enforcement is important for three reasons. First, adolescents may make use of staff members who do not strictly enforce the smoking ban by using them as opportunities to smoke. Second, staff members’ inconsistent enforcement may lead adolescents to perceive the smoking ban as unfair (e.g. different sanctions applied to different adolescents). Third, adolescents may start rebelling against the school’s authority when the rules are perceived to be inconsistent.

While staff enforcement is important for the effectiveness of STPs, there is only a limited understanding of what determines the consistency of staff members in terms of STP enforcement. Research has demonstrated a connection between staff members’ responses to STPs and the staff’s actual enforcement behaviour. For instance, Gordon and Turner’s [11] study showed that the perceived effectiveness of STPs combined with the staff’s personal and professional values, sense of authority, and perceived issues regarding their own safety influenced the staff’s STP enforcement. These responses, in turn, likely depend on differences in context. A realist review [12] on the implementation of health promotion programmes in schools showed how the responses of staff members that are needed for adequate implementation depend on different school-level contextual factors. For example, teachers are more likely to devote their time and energy to programme implementation if they believe that they will get practical and educational support.

Most of the current literature on STP enforcement by staff members report either on the context or on the responses, but how these factors are connected is rarely explained. Our realist review will contribute to this gap in the current understanding by explaining how staff members’ responses, which make up their STP enforcement, differ across contexts. The realist review is a suitable method, because it aims to explain how contextual factors (in our case, at the individual, interpersonal, school, implementation, and national levels) produce outcomes (in our case, staff enforcement) by specifying the underlying generative mechanisms (in our case, the staff’s cognitive, psychological, and behavioural responses) [13]. We aim to draw together existing evidence and build an evidence-based programme theory that answers the following question:

1. How do contextual factors at the individual, interpersonal, school, implementation, and national levels (Context) contribute to triggering staff members’ cognitive, psychosocial, and behavioural responses (Mechanism) that may support their STP enforcement (Outcome)?

## Methods

A realist review is an explanatory method that aims to describe what works for whom, under what circumstances, and how. It synthesizes evidence into a programme theory explaining how differences in contexts may lead to outcomes by forming the enabling conditions that allow generative mechanisms to occur [13]. The generative mechanisms are the underlying processes or hidden causal levers that account for how and why policies or programmes work to bring about changes in the reasoning and behaviour of individuals [14]. The realist review consists of six iterative steps: (1) identifying the review questions; (2) formulating the initial programme theory; (3) searching for primary studies; (4) selecting and appraising the studies; (5) extracting, analyzing, and synthesizing relevant data; and (6) refining the programme theory [13]. Step 1 was done in the “Background” section above, and the remaining steps are reported below. We followed the RAMESES publication standards for realist reviews [15].

### Formulating the initial programme theory

The initial programme theory (Table 1)—i.e. the initial understanding of the CMO configurations—was formulated between January and March of 2016. To build up the initial programme theory, we first read recent literature reviews on STPs [1, 7, 8, 16]; policy reports and guidelines for STP implementation from Finland, which has a long tradition in implementing these policies [17–21]; and a review on the implementation of Health Promoting Schools (HPS) [22]. These steps assisted us in identifying the relevant contextual factors. However, the above-mentioned materials did not provide us with sufficient understanding of the mechanisms that might occur and require further testing. Therefore, we interviewed people who had significant work experience in the implementation of STPs: an expert from the Finnish National Institute for Health and Welfare, a school principal, and three teachers from different schools. The interviews helped us to gain an understanding of what possible mechanisms may connect the identified contexts with staff enforcement. The RAMESES guidelines recommend the use of both scientific literature and expert experiences for the development of an initial programme theory [15]. Table 1 presents this initial programme theory.

**Table 1:** Initial programme theory explaining how contextual factors may trigger mechanisms that influence staff’s STP enforcement

CMO1: Alignment of staff and overall health promoting culture in the school (C), trigger staff’s acceptance and readiness for STP enforcement (M), which may lead to staff members’ STP enforcement (O)	
CMO2: Inclusion of comprehensive and consistent STPs in school policy document that are packed up by legislation (C), trigger priority of abstinence from smoking at school and staff’s significant role in ensuring that (M), which may lead to staff members’ STP enforcement (O)	
CMO3: Supportive leadership and management (e.g. senior management’s actions) (C), trigger shared values and motivation for tobacco-free school among staff (M), which may lead to staff members’ STP enforcement (O)	
CMO4: Continuous and sustainable focus on STPs and other health issues in school (C), trigger changes in school smoking norms (M), which may lead to staff members’ continuous STP enforcement (O)	

### Searching for primary studies

Next, a systematic literature search was conducted to refine and substantiate the CMOs in the initial programme theory. The systematic search included two separate search strategies (Additional file 1) that were refined in collaboration with information specialists. The two searches were used to generate understanding about STP implementation (search strategy 1), and the implementation of health promotion in schools (search strategy 2). The second strategy was conducted because there is scarce literature on STP implementation, while there is a wealth of literature on the implementation of Health Promoting School concept (HPS) and school health promotion programmes. HPS concept and school health promotion programmes share the same setting and implementation processes, with staff members as key actors, and they therefore provide valuable evidence for refining and substantiating the CMOs in our initial programme theory. Searches were conducted using multiple databases from diverse disciplines (e.g. social sciences, psychology, education, health policy, and health sciences; see Additional file 1). The language was limited to English and the timeline was from January 2000 to March 2016. We chose to include articles only from 2000 onwards because of the large number of publications.

### Selecting studies and appraising their quality

**Fig. 1:**
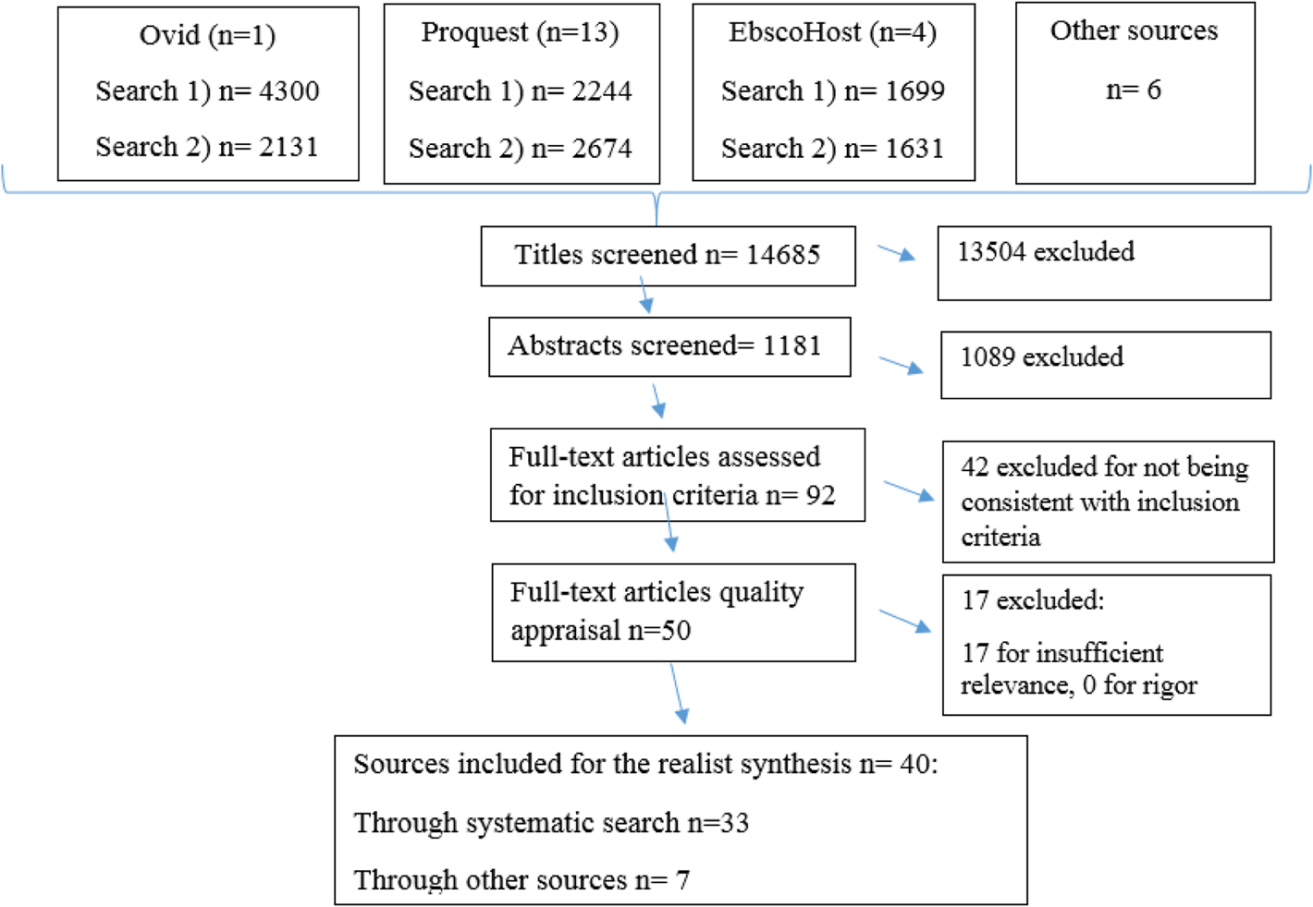
Flow diagram representing the search, screening, and inclusion of the articles

Figure 1 depicts the flow diagram of the searches and selection of the articles. Altogether, 14,685 unique articles were found. From these articles, we first screened titles and abstracts. To be selected further, an article had to provide information on one or more of the following themes: (1) STP implementation, (2) implementation of health promotion in schools, (3) mechanisms explaining staff members’ perceptions and behaviour, (4) information on the school as a context, or (5) other contextual factors influencing STP enforcement in schools. Ninety-two full-text articles were selected for further assessment after screening the titles and abstracts. Next, the same inclusion and exclusion criteria were applied to these 92 articles, and this process yielded 50 articles.

Next to articles on STP enforcement, we also included articles on STP adoption (i.e. the decision-making process to implement STPs) because implementation and adoption are not categorically distinct processes.

From the remaining 50 articles, we highlighted the relevant pieces of evidence for our study, and following the RAMESES publication standards for realist reviews, a quality appraisal for relevance and rigour was made for these selected pieces of evidence [15] (Additional file 3). The relevance of the articles with the selected pieces of evidence was assessed according to the extent to which they refined, confirmed, or added to the initial programme theory. Articles with a select piece of evidence that provided in-depth information on mechanisms were defined as “thick”. Articles that did not describe mechanisms but provided other relevant information—e.g. understanding on context—were defined as “thin”. Seventeen articles did not provide “thick” or “thin” evidence, and they were therefore excluded.

The rigour of the selected pieces of evidence was assessed by examining each article’s sample, data collection, and methods of analysis before determining how these features might affect the validity of the evidence. The quality appraisal of rigour was conducted as the last of all the steps; none of the remaining articles was excluded at this point. Two authors (AL, PL) worked together during the selection and appraisal of the studies, and all articles included in the final synthesis were examined and approved by both authors. In addition to the evidence found through the systematic search, seven articles published before or after the timeline of the search were included, as they provided valuable evidence for analysis. These articles were found through citation searching from the articles included and manual searches of the latest publications. The final number of articles was forty. Additional file 2 outlines the key characteristics of the selected studies.

### Extracting, analyzing, and synthesizing relevant data

The first author extracted the relevant pieces of evidence from the selected articles in order to refine or substantiate the initial programme theory. We began the synthesis with the “thick” evidence by studying how contexts, mechanisms, and the staff members’ enforcement are connected. Finally, the CMO configurations were compared to the thinner evidence to reveal further contextual refinements. The findings were reflected upon by the authors for a period of 1 month. All authors approved the final programme theory.

## Results

We revised the initial programme theory into a refined programme theory. The refined programme theory revised the initial outcome “staff members’ STP enforcement” into three sub-outcomes: responsibility, motivation, and confidence in STP enforcement. In addition, the refined programme theory includes many new contextual factors (see Table 2).

**Table 2:** Refined program theory explaining how factors at different contextual levels may trigger mechanisms that influence staff’s responsibility, motivation and confidence for STP enforcement

	Factors at different contextual levels	Mechanism	Outcome
CMO1	• Individual Staff’s professional identity and values, e.g. health promotion vs academic education	Staff experience STPs part of the school staff’s professional role and duties	Responsibility for STP enforcement
	• Interpersonal Staff’s perceptions on the influence of enforcement to staff-student relationships		
	• School Existing workload and the significance of tobacco issue in school		
	• Implementation components Anchoring and communicating STP, including staff abstinence from smoking during school hours, as part of the school’s core tasks and all staff’s role and duties through written policies and senior management’s engagement; come up with enforcement practices that do not threaten staff-student relationships		
	• National Legislation on STP and on tobacco in wider environment, e.g. ban on smoking in public places		
CMO2	• Individual Staff’s perceptions on schools general ability to influence student smoking Student’s characteristics (e.g. nicotine addiction)	Staff perceive that their contribution is leading to positive outcomes	Motivation for STP enforcement
	• Interpersonal Other staff members’ participation to the enforcement		
	• Implementation components Coming up with strategies to tackle enforcement problems (e.g. smoking relocation that increase visibility, non-effective enforcement practices for students with nicotine addiction) e.g. to avert inconsistent enforcement among staff, communicating staff about the progress achieved with STP		
	• National Conformity in tobacco norms and aims between school and wider society (i.e. back up for STP)		
CMO3	• Individual Staff member’s own smoking status Student’s characteristics (e.g. physical or verbal aggression)	Staff feel that they are able to deal with students’ responses	Confidence for STP enforcement
	• Interpersonal Staff’s familiarity with the student		
	• Implementation components Communicating all staff’s authority for STP enforcement and strengthening staff members’ skills to enforce with difficult or unfamiliar students		
	• National Legislation on STP and on tobacco in wider environment, e.g. ban on smoking in public places		

The refined programme theory features three CMOs: when contextual factors make staff members experience STP as part of the school staff’s professional role and duties, it may lead to staff members showing responsibility for STPs enforcement (CMO1); when contextual factors make staff members feel their contribution is leading to positive outcomes, it may lead to staff members showing motivation for STP enforcement (CMO2); and when contextual factors make staff members feel that they are able to deal with students’ responses, it may lead to staff members showing confidence for STP enforcement (CMO3). The remainder of the “Results” section elucidates each of these CMOs using the evidence that was found during the synthesis.

### CMO1: When contextual factors (C) make staff experience STP as part of the school staff’s professional role and duties (M), it may lead to staff members showing responsibility for STP enforcement (O)

Staff members tend to commit to enforcement only when they know what is expected of them—i.e. they know what their duties are [12, 22–26]. Gordon and Turner [11] found that uncertainty regarding whether intervention in student smoking was expected or simply desirable in school policies led to variations in the behaviour of school staff. Therefore, anchoring health promotion and STPs in school policies as well as clearly communicating the staff members’ duties in STP enforcement—preferably through written policy—may remove any ambiguity as to what is expected, and it may increase the staff’s responsibility for enforcement [11, 22, 26–33].

In addition to written policies, the senior management’s role in outlining the school values and policies and directing the enforcement is emphasized [12, 25, 29, 30, 33, 34]. For instance, the senior management’s commitment to STPs may affect the staff members’ perception of the policy’s importance [12, 25, 29, 30, 33, 34] and further promote the staff’s responsibility for enforcement [11, 22, 31, 35]. Conversely, STP enforcement is perceived of as challenging when the senior management is not engaged [25, 29].

Staff members who perceive that health promotion—e.g. protecting students from the harmful effects of smoking—is compatible with their professional identity, and values show more responsibility for STP enforcement [5, 23, 36]. On the other hand, those staff members who do not consider health promotion a professional duty and have a “philosophical resistance” to modifying adolescents’ health behaviour show less responsibility for intervening in student smoking [11, 23, 24, 37–39]. Some staff members may not consider STP enforcement their duty because they think it distracts from the core task of education: “People forget that we’re a school, focusing on the education of students” [23].

Staff members’ perceptions of whether their own smoking influences student smoking may have an impact on the staff members’ responsibility for STP enforcement. When staff members consider themselves non-smoking role models for students, they may also acknowledge STPs as part of the school’s core task [40–42]: “We made people (staff members) aware of what kinds of messages we’re sending to our children through tobacco use. When you put it in light of the youths, people are willing to comply” [40]. Furthermore, when staff members perceive that students support STPs and expect staff to set an example, it may reinforce their responsibility and enforcement: “Hearing from students was the most effective, hearing from them that adults and schools should be setting examples” [40].

National legislation on STPs could be utilized to strengthen the staff’s responsibility for STP enforcement. When national legislation compels schools to enforce STPs, the mandatory nature of the laws may make staff members feel more responsible for enforcement [33, 43, 44]. Other tobacco legislation, such as smoking bans in public places, may also increase the acceptance of STPs and in this way increase the staff members’ responsibility for enforcement [11, 45–49].

The staff’s perceptions of the influence of STP enforcement on staff-student relationships may affect feelings of responsibility for enforcement. If intervening in student smoking clashes with the type of relationship staff members wish to have with students, or which the staff perceive to be the basis for effective collaboration, the inconsistency may lead to prioritizing good relationships over enforcing STPs: “You don’t learn to manage them [pupils] by creating lots of rules and making sure that you enforce them. You manage them by establishing the relationship and working with the child, not against the child, and through that relationship to an understanding that there is a way of working together which is in both our interests. And that would be my attitude towards smoking.” [11].

School working conditions, like overwork and smoking not being considered a priority health issue, may influence the staff’s responsibility for STP enforcement. For instance, perceptions of responsibility may decrease when the school is burdened with other tasks [11, 23, 24, 36, 43] or when other health issues are considered a greater priority [11, 43, 50]: “If you were going to really look at what the health issues are, smoking isn’t the most important one” [43].

### CMO2: When contextual factors (C) make staff perceive that their contribution is leading to positive outcomes (M), it may lead to staff members showing motivation for STP enforcement (O)

When staff members believe that STP have positive outcomes for the school, staff, or students, it may trigger their motivation for STP enforcement [12, 23, 24, 32, 51, 52]. Staff members’ perceptions of whether the school is generally able to influence adolescent smoking may affect their outcome expectations and motivation for enforcement [11, 50]. For instance, if staff members think that peers, parents, and social norms play a more significant role in adolescent smoking than the school, it may compromise their motivation to enforce STPs [11, 45–49].

The behaviour of colleagues is one factor that may influence staff members’ perceptions of the ability of STPs to make an impact. If staff members witness their colleagues turning a blind eye to student smoking, their positive expectations of being able to make an impact on students—and thus their motivation to enforce STPs—may decrease [11, 23, 31]. Staff considered the participation of all personnel—i.e. senior management, teaching staff, and non-teaching staff—in STP enforcement to be crucial in influencing student smoking and normalizing smoking bans as a part of the school culture (i.e. becoming a tobacco-free school) [31, 53].

In addition, the characteristics of the smoking student may influence the staff members’ expectations on enforcing STPs. For instance, recognizing a student’s tobacco addiction may cause a contradiction with the staff members’ motivation to enforce STPs, because intervening could be considered “fire-fighting” rather than solving the smoking problem [49]. The school policy on the consequences of breaking the smoking ban was particularly important when dealing with addicted students, as staff members preferred supportive rather than punitive measures [29, 43, 49].

Pearson et al.’s [12] study showed that beliefs about policy effectiveness may change during the implementation process when the positive results are witnessed and valued. Pickett’s [48] study showed that staff members’ support for the policy increased when they witnessed a decrease in student smoking after implementing the smoking ban. Conversely, when the ban was considered ineffective, a return to designated smoking areas received support from staff members [48]. Therefore, schools may increase staff members’ positive outcome expectations and thus motivation for STP enforcement through consistent practices like monitoring, evaluating, and communicating the improvements and effectiveness of STPs [29, 38, 54–58].

STP enforcement may also have negative outcomes that influence the staff’s attitudes towards STPs and their motivation for enforcement [24, 25, 29, 33, 40, 42, 51, 59]. Smoking relocation (e.g. from hidden smoking places to the boundaries of the school) was the most often reported negative outcome of STP enforcement, which also decreased the positive outcome expectations of the effectiveness of STPs [29, 43]. The relocation of smoking often increased the visibility of smoking, which staff considered harmful for the de-normalization of smoking in the school [24, 31] and for the school’s image, and this therefore influenced the staff’s motivation to enforce the STPs [24, 29, 46]: “We’d rather have people hidden at a couple of places throughout the campus than have a large group of smokers as the first thing people see when they arrive” [29]. Furthermore, the relocation of smoking caused a nuisance to the school’s neighbours [24, 29, 43, 47] and raised concerns over safety when students left the school grounds to smoke [27, 29, 46–48, 53].

The national context may also play a part in smoking relocation in schools. Existing legislation rarely prohibits smoking in school surroundings, and thus it restricts and sets limits on the staff members’ jurisdiction [12, 22–26]. The lack of rules and legal authority to intervene demotivate staff members to enforce the STPs, because the outcomes of the enforcement are negative and visible: “It is legal for kids to smoke on public property, whether that property is one inch or one mile away from school property” [46]. Furthermore, the school may not be entitled to issue sanctions for smoking outside school grounds [11, 29, 46, 47], which the students are also aware of: “they’re just going to tell me, ‘you’re nothing to do with me’, you know they’re not in the school...” [11]. This limited authority to intervene, even when smoking is clearly visible just outside the school premises, decreased the motivation of staff to enforce STPs [11, 29, 46, 47]. One way to tackle smoking relocation is, for instance, to prohibit students from leaving the school grounds during the school day [11, 31].

### CMO3: When contextual factors (C) make staff feel that they are able to deal with students’ responses (M), it may lead to staff members showing confidence for STP enforcement (O)

Staff commit to enforcement when they feel confident enough to intervene in student smoking [12, 22–26]. This level of confidence, in turn, depends considerably on the staff members’ feelings about their ability—e.g. skills—to deal with the adolescents’ responses. The characteristics of students influence the staff members’ perceptions of their ability and thus confidence to enforce the STPs, as smoking students were sometimes perceived of as being dismissive of the staff members’ authority or indifferent to the consequences of getting caught [49]. Staff were also discouraged from intervening if they expected the student might be threatening [11, 47]. In addition, sometimes the staff members’ lack of familiarity with a student decreased their ability to strictly intervene in smoking: “the pupils’ lives can be so complicated and me just coming in there and giving them a row for smoking might be so trivial compared to what’s going on in their house” [11].

The staff members’ own personal smoking habits may also decrease their ability—e.g. authority—to intervene in student smoking. Staff members who smoke may feel that they are not fully entitled to take action against student smoking, and students may use the staff member’s smoking as an argument against enforcement [28, 29, 31].

At the national level, legislation compelling schools to implement STP strengthens staff’s abilities to intervene in student smoking, because government rules stand as a backbone and give staff authority for enforcing with criticizing students [6, 29, 43]. Legislation may also indirectly decrease the students’ negative responses, as legislation on smoking bans in society (e.g. restaurants, bars, workplaces) gradually de-normalize smoking, which may make staff intervening in student smoking acceptable and expected behaviour [24, 29, 51, 53, 59].

## Discussion

The purpose of our realist review was to improve our understanding of why staff members in some schools enforce STPs more consistently than others by explicating how contextual factors at the individual, interpersonal, school, implementation, and national levels contribute to triggering school staff members’ cognitive, psychosocial, and behavioural responses (mechanism), which may in turn influence their enforcement behaviour (outcome). We discovered three generative mechanisms, which we integrated into a programme theory.

CMO1: When contextual factors make staff experience STP as part of their professional role and duties, it may lead staff members’ responsibility for STP enforcement. Key contextual factors that may trigger responsibility are the staff members’ professional identity and values (e.g. they appreciate school health promotion) and perceptions that enforcement does not considerably burden them or negatively influence staff-student relationships.

CMO2: When contextual factors make staff perceive that their contribution is leading to positive outcomes, it may lead to staff’s motivation for STP enforcement. Key contextual factors that may trigger motivation are the staff members’ perception that schools can compensate for negative peer and family influences and their perception that all colleagues are doing their part and participating in enforcement.

CMO3: When contextual factors make staff feel that they are able to deal with students’ responses, it may lead to staff’s confidence for STP enforcement. Key contextual factors are the staff members’ own smoking status, non-familiarity with students, and the expectation that students will respond aggressively. Although the programme theory presents the CMOs separately, they are interconnected, as the staff’s responsibility (CMO1) and confidence in STP enforcement (CMO3) influence the consistency of all staff members in enforcing STPs. This further triggers the staff’s outcome expectations and motivation for STP enforcement (CMO2).

This was the second realist review looking at how to facilitate staff members’ implementation of health promotion policies/programmes in the school context. Pearson et al.’s [12] review showed how the implementation of programmes in schools could be supported by focusing on contextual factors at the school level. Our review extends this work by demonstrating that it is also important to examine and address the influence of contextual factors beyond the school level when aiming to understand and improve the staff’s implementation. The school is thus not the only stakeholder that should be held accountable for safeguarding the effective implementation of school health promotion policies/programmes.

Prior studies examining programme implementation in schools have pointed out that support from senior management is a key element in successful implementation, yet they did not explicate in detail why this is the case [12, 60]. Our results indicate that this support is important because the senior management plays a central role in developing the school culture, practices, and values that influence the staff’s feeling of responsibility for enforcement (CMO1). Individual teachers or groups of teachers can act as champions for health promotion, but in a long run, they also need support from the senior management. Based on related literature in health care settings [61], we also expect that support from the senior management influences staff’s expectations of a positive outcome (CMO2) and staff’s feeling of confidence (CMO3). Senior management could, for instance, deal with students who disrespect an intervention by a staff member who is lower down in the hierarchy, therewith increasing staff’s confidence to intervene.

A novel finding was the importance of staff members’ collective STP enforcement for individual staff members’ expectations of a positive outcome. Earlier research on STPs had already demonstrated that the consistency of staff members’ enforcement influences the impact of STPs on adolescent smoking [7, 8, 10], and our results explain this by showing that colleagues who turn a blind eye to student smoking compromise other staff members’ expectations of a positive outcome and consequently the motivation for enforcement (CMO2). This explanation fits with the normalization process theory [62], which underlines that the contribution of all staff is important for policies to become embedded in specific social contexts. Our results can also be reflected in the theoretical framework of schools as complex adaptive systems (CAS) [56] to highlight how each staff member’s behaviour influences the school dynamics and vice versa.

### Practical recommendations

The results show that staff members’ positive outcomes may decrease if implementing and enforcing smoking bans on the school premises leads to smoking outside the school’s boundaries and increased smoking visibility. A solution at the school level could be to prohibit students from leaving the grounds during school hours—that is, making the school hours a smoke-free time for all adolescents. However, one may question whether such a rule would not lead to adverse effects for the most vulnerable students. A more feasible solution may be to adopt a government policy that permits schools to enforce the smoking rules during school hours outside the areas that fall under schools’ formal jurisdiction.

The results also demonstrate the significance of staff members’ collective STP enforcement, highlighting the importance for schools to engage all staff members in the enforcement of STPs. There were many reasons explaining why individual staff members may not enforce the rules, but one important reason was that staff members question the impact of punitive sanctions for nicotine dependent students. Therefore, it is important for schools to find ways to support nicotine-dependent adolescents; otherwise, staff members will remain reluctant to enforce the rules, in turn, decreasing the overall impact of the STP [10].

Schools should be motivated not only to aim at promoting adolescents’ academic outcomes, but also to contribute to the students’ overall health and well-being. The results indicate that national policies have an important role to play in making staff members feel that STP enforcement—and health promotion more generally—is part of their professional role. Finland is an example of a country where national laws on education and health presume co-operation between sectors in the education and welfare activities of schools. A basic education law [63] also aims to promote student health and well-being and to develop a school culture that promotes both learning and well-being. Furthermore, a specific law [64] stipulates school and student welfare activities. The ability of schools to apply and integrate interventions and health promotion programmes in their basic activities in a way that generates permanent effects is nationally monitored on a regular basis [65].

### Limitations and future research

The programme theory explains under what conditions staff members feel responsible, motivated, or confident (i.e. generative mechanisms) to enforce STPs. However, the main limitation is that the programme theory is unable to differentiate the relative influence of the contextual factors and generative mechanisms on actual staff members’ enforcement behaviour. Such questions of relative influence are best addressed in future studies using quantitative methodologies.

Another limitation is that evidence on contextual factors at the school and intrapersonal levels was scarce. Future research should focus in more detail on these school and intrapersonal level factors, because they are likely easier to tackle by schools and local stakeholders compared to national-level factors. For instance, our results show that staff experience difficulties in enforcing STPs when they know students are addicted to nicotine, yet evidence on possible solutions to this problem remains absent.

## Conclusions

By applying a realist approach, we have been able to detect three CMOs that explain school staff members’ STP enforcement. We have extended the contemporary understanding of the complexity of implementation in the school context by thoroughly specifying how contextual factors at different levels (e.g. the individual, interpersonal, school, implementation, and national) may influence staff members’ STP enforcement. The study offers insights for policymakers and stakeholders on how to support staff members’ STP enforcement and thereby the effectiveness of STPs on adolescent smoking.

## Additional files

Additional file 1: Search strategy. (DOCX 155 kb)

Additional file 2: Main characteristics of the included studies. (DOCX 36 kb)

Additional file 3: RAMESES publication standards: realist syntheses. (DOCX 15 kb)

## Data Availability

The dataset supporting the conclusion of the article is included within the article. Consultative interviews in the beginning of the review process were not recorded nor archived systematically.

## Abbreviations

HPS: Health Promoting School
MEDLINE: US National Library of Medicine premier bibliographic database
PsycInfo: Resource for abstracts and citations of behavioural and social science research
RAMESES: Realist and Meta-narrative Evidence Syntheses
RR: Realist review
STP: School tobacco policies
