# Supplementary material for: Understanding school staff members’ enforcement of school tobacco policies to achieve tobacco-free school: a realist review
Source: Syst Rev. 2019 Jul 19;8:177. doi: 10.1186/s13643-019-1086-5 (PMC6642528; doi:10.1186/s13643-019-1086-5)
Supplement: Supplementary file 2 — Main characteristics of the included studies. (DOCX 36 kb) [file 13643_2019_1086_MOESM2_ESM.docx]

**Understanding school staff members’ enforcement of school tobacco policies to achieve tobacco-free school: a realist review.**

Linnansaari Anu^1^, Schreuders Michael^2^, Kunst Anton^2^, Rimpelä Arja^1,3,4^, Lindfors Pirjo^1,3*^.

1. Faculty of Social Sciences, Health Sciences, P.O. Box 100, 33014 Tampere University, Tampere, Finland
2. Department of Public Health, Amsterdam UMC, University of Amsterdam, Amsterdam Public Health Institute, Amsterdam, The Netherlands.
3. PERLA—Tampere Centre for Childhood, Youth and Family Research, 33014 Tampere University, Finland
4. Department of Adolescent Psychiatry, Pitkäniemi Hospital, Tampere University Hospital, 33380 Nokia, Finland

[anu.linnansaari@tuni.fi](mailto:anu.linnansaari@tuni.fi)

[m.schreuders@amc.uva.nl](mailto:m.schreuders@amc.uva.nl)

[a.kunst@amc.uva.nl](mailto:a.kunst@amc.uva.nl)

[arja.rimpela@tuni.fi](mailto:arja.rimpela@tuni.fi)

[pirjo.lindfors@tuni.fi](mailto:pirjo.lindfors@tuni.fi%20)

* Corresponding author
